# Numerical Modelling of the Constitutive Behaviour of FRCM Composites through the Use of Truss Elements

**DOI:** 10.3390/ma16031011

**Published:** 2023-01-22

**Authors:** Maria Concetta Oddo, Giovanni Minafó, Marielisa Di Leto, Lidia La Mendola

**Affiliations:** Department of Engineering, University of Palermo, Viale delle Scienze, 90128 Palermo, Italy

**Keywords:** FRCM, masonry, modeling, tensile behavior, shear bond behavior

## Abstract

The modeling of the mechanical behavior of Fabric Reinforced Cementitious Matrix (FRCM) composites is a difficult task due to the complex mechanisms established at the fibre-matrix and composite-support interface level. Recently, several modeling approaches have been proposed to simulate the mechanical response of FRCM strengthening systems, however a simple and reliable procedure is still missing. In this paper, two simplified numerical models are proposed to simulate the tensile and shear bond behavior of FRCM composites. Both models take advantage of truss and non-linear spring elements to simulate the material components and the interface. The proposed approach enables us to deduce the global mechanical response in terms of stress-strain or stress-slip relations. The accuracy of the proposed models is validated against the experimental benchmarks available in the literature.

## 1. Introduction

The use of Fabric Reinforced Cementitious Matrix (FRCM) materials is today a very common practice in the field of structural retrofitting of existing buildings. The FRCM composites consist of fiber grids of various nature (e.g., basalt, glass, carbon, polyparaphenylene benzobisoxazole, aramid) embedded between two layers of mortar and are based on lime or cement binders. Their popularity is mainly due to the use of the inorganic matrix that provides an improved compatibility with the stone and masonry support [1,2] compared to the well-known Fiber Reinforced Polymers (FRPs). It should be noted that Naser et al. [3] stressed that in the presence of different kinds of loads, it is of fundamental importance to model the constitutive bond behavior of the matrix-to-support interface and to address its dependence on the materials that it comes into contact with.

Recently, the technical community has increased its interest towards this new emerging class of composite materials as demonstrated by the rapid development of recent International Standards, which aim to define the experimental procedures and reliable methods for assessing the constitutive behavior of FRCM materials. The provisions reported in these standards are the summary of a large amount of experimental work developed in recent years, which aimed to investigate the role of all the test variables on the mechanical behavior of FRCM composites.

The focus of the research on these materials, and generally on Fiber Reinforced Concrete (FRC) materials, is on the influence of the fiber’s characteristic properties. In Shi et al. [4], it is shown how the fibers clearly affect the matrix post-cracking behavior. While Bai et al. [5] analytically studied how the tensile behavior changes as the fiber content changes, as well as the effects of this parameter on the initiation and expansion of microcracks. Studies have also been conducted on the response of FRCM material in presence of different environmental conditions [6], effects of rising damp and salt crystallization cycles [7] and with the effect of the wet-dry cycle [8]. These also show how the external conditions influence the breaking modality and the debonding mechanisms of the interface. Many studies presented in the literature show how, in the presence of a great variability of constituent materials, each FRCM system needs its own study, according to its unique properties. It is possible to find a summary in the study proposed by Hojdys and Krajewski [9], in which six different samples are analyzed, their different properties are studied, the results are compared, and particular attention is also paid to the influence of any accidental load eccentricities. It is therefore clear how complex it is to analyze the response of these materials and how fundamentally important it is to be able to find simplified models that can reduce the computational effort.

Despite their growing spread, the current procedures for the mechanical characterization of these composites are still under discussions. Likewise, few modeling approaches were developed in the literature for representing the constitutive behavior of FRCM materials.

Generally, the available theoretical studies are based on detailed finite element (FE) models or micromodels and simplified analytical approaches. However, a reference study for a simple yet accurate numerical modeling method is still missing or under study.

Simplified analytical formulations proposed in the past [10,11] were revisited with the aim to predict the typical tri-linear tensile behavior of the composite systems. They are based on the well-know rules adopted for reinforced concrete members: Aveston-Cooper-Kelly (ACK) Model [12], model proposed by Minafò and La Mendola [11] and Tension Stiffening Model [13]. However, a study of review [14], highlighted that the aforementioned analytical models allow an estimate the tensile behavior of the FRCM systems with good enough accuracy but with several limitations. Indeed, the three models are unable to take into account the influence of the complex mechanisms of interaction between the fiber textile and the mortar. In the literature, it is also possible to find several refined theoretical studies. Examples are those proposed by Grande and Milani [15] and Misseri et al. [16], which consider different constitutive laws of the fiber-matrix interface for numerical simulations. Grande and Milani [15] proposed a procedure for calculating shear stress-slip laws for simulating the local bond behavior of FRCM systems at the reinforcement/matrix interface by combining experimental results with the equations of a proposed model. While Misseri et al. [16] attribute different cohesive material laws to the interface for an assumed value of fracture energy representative of the material system and studied the effect that the different laws have on the global response. Other works based on numerical approaches, i.e., 3D or 2D FE models [17] and non-linear micro-mechanical models [18], are proposed. An accurate response is obtained from the use of the multi-scale approach for modeling the bond behavior between the FRCM system and the strengthening support [19] and the FRCM tensile behavior [17,20]. In these cases, each constituent material and interface level is modeled separately with the most suitable finite elements. The models provide quite good prediction of the global response and the local non-linear behavior at the interface level, simulating the crack pattern well. However, these modeling approaches require long computational time, as well as convergence issues. Moreover, these models need the definition of variables that are often unknown and require to be experimentally calibrated or tentatively estimated, resulting in some unreliability. An example is given by Shah et al. [21], who propose a multi-scale homogenization for the prediction of the elastic constants of FRC composites and highlight the complexity encountered both in experimentally determining the variables and in predicting them with numerical or analytical methods. More recent studies proposed uniaxial models and numerical strategies to solve the governing equations of FRCM strips under different loading conditions. In particular, Grande and Milani (2020) [22] proposed a procedure to solve a system of differential equations derived by equilibrium considerations involving the main components of a FRCM system, i.e., fiber, mortar and the fiber-mortar interface, where specific constitutive laws and boundary conditions, as well as accounting for the occurrence of damage, are introduced. This approach demonstrates a certain reliability in predicting the experimental response, emphasizing the important aspects concerning the local behavior of FRCM systems in the presence of possible softening behaviors of both the mortar and the fiber-mortar interface.

A 3D FE model was proposed by Mazzucco et al. [23] that uses a meso-scale approach, taking into account the matrix-fiber adhesion behavior and matrix-textile interlocking. Carozzi et al. [24] adopt a non-linear FEM approach and employ 8-noded rigid elements interconnected by inelastic interfaces exhibiting softening, while a variational model was created by Donnini and Corinaldesi [25] to simulate the debonding process and the slippage at the yarn-to-matrix interface, paying particular attention to the interface energy formulation.

On these bases, the proposed work aims to contribute to the advancement of the knowledge pertaining to the mechanical behavior of FRCM composites for structural retrofitting applications by thoroughly studying their constitutive characterization and the bond behavior from a numerical point of view. For the scope, two numerical models are developed to predict the tensile and the shear bond behavior by exploiting a simplified FE approach. Both models are developed with the aim of reducing the computational effort and providing an easily reproducible and implementable model by using simple truss elements and shear zero-thickness discrete interface elements. With the adoption of this approach, combined with a careful evaluation of the interface constitutive law, we demonstrate that we can predict the experimental response of the FRCM systems under tension.

## 2. Simplified Modeling Approach

Two 1D numerical models are proposed to simulate the tensile and shear bond behavior of FRCM composites, respectively. Both models take advantage of truss and non-linear spring elements to simulate the material components and the interface. The implementation is completed through OpenseesPy [26], which is a Python interpreter of the FE opensource framework OpenSees [27].

### 2.1. Tensile 1D Model

The proposed model is developed for simulating the mechanical response of FRCM strips subjected to a tensile load, adopting the clamping grip method. The main phases (i.e., fabric and matrix) are modeled with two orders of truss elements, one for mortar and one for fabric, respectively. Additionally, axial spring elements are added in series at the ends of each matrix truss. These springs, called “brittle hinges”, are introduced in order to simulate crack opening in the mortar. Moreover, shear spring elements connect the mortar elements to the row of fabric trusses in order to simulate the fabric-to-matrix interaction. It is worth pointing out that the proposed simplified model neglected the inclusion of multiple random fibers because the available literature highlights that the main reinforcement layer consists of a bidirectional fiber grid stressed in the axial direction. Thus, fabric truss elements simulate only the longitudinal bundles. This assumption is coherent in the experimental observations available in the literature [28]. Bellini et al. [28] demonstrated that, during tensile tests, transversal bundles of textiles are embedded exactly in their original position, still wrapped by the two matrix layers when the failure occurred.

The model is developed under the following hypotheses:

1. the fiber fabric is considered to be a bidirectional grid and only the fibers parallel to the load are considered to carry the load;
2. geometrical imperfections are neglected (i.e., the FRCM strip has a straight axis);
3. both phases behave only in pure tension;
4. the first crack opens at the member’s ends;
5. the fabric-to-matrix interface can transfer pure shear stress.

The assumptions made are simplified, but they allow for a model that is simple and easily implemented, with a limited number of parameters to be calibrated and reduced computational effort. The validity of these assumptions is warranted by a broad experimental validation and can be tested by comparing the results with those obtained through more refined approaches proposed in the literature [17,18,19,23].

**Figure 1:**
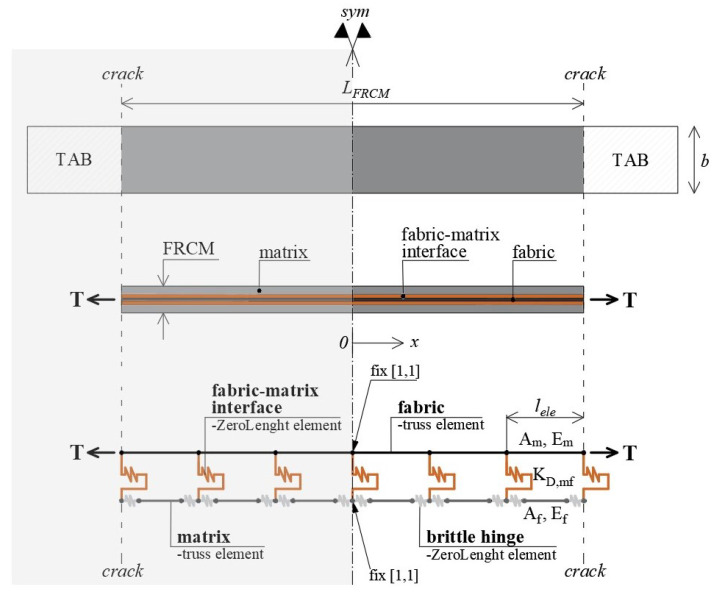
1D numerical models for tensile behavior.

On the basis of these hypotheses, the model is defined along the axis of the FRCM strips, and therefore it can be defined as a uniaxial model. The proposed model takes advantage of the symmetry condition, hence it is possible to study half of the FRCM strip, fixing the degrees of freedom of the nodes at the center axis, about which the system is symmetrical, as depicted in Figure 1.

**Figure 2:**
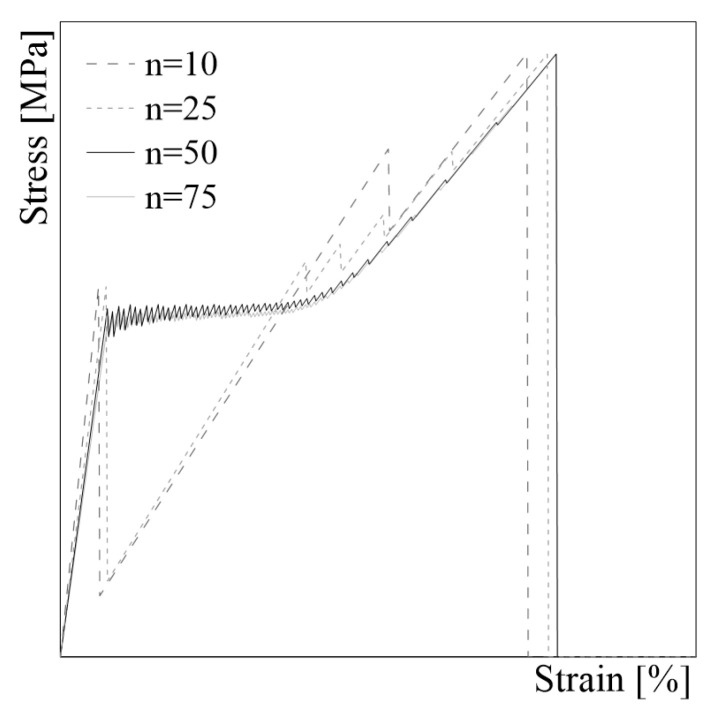
Mesh sensitivity test.

The number of elements is assumed in order to achieve a suitable numerical response. Numerically, it is observed that the amplitude of each load drop decreases when the number of nodes increases. The numerical response as a function of the number of elements *n* for a generic sample is shown in Figure 2. When the model is discretized in more than 50 elements, the trends of the curves do not change significantly, indicating that the model has achieved stability, as well as that the refined mesh discretization has little impact on the numerical outcomes.

**Figure 3:**
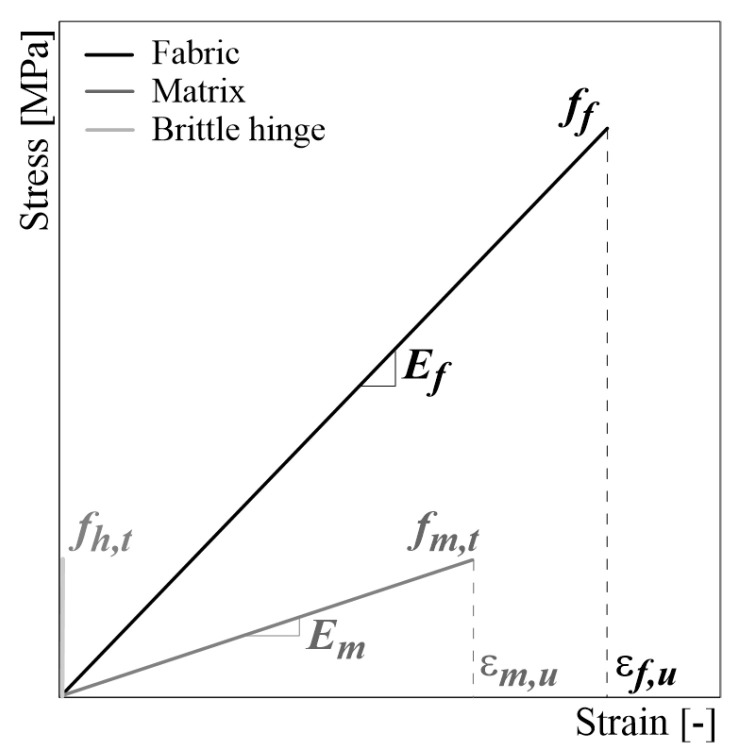
Constitutive laws assumed for material constituents.

In the present work, the mesh size is calibrated by assuming an element dimension of 5 mm. This assumption ensures a reliable response with minimal computational effort. The two series of truss elements are assumed to be elastic with a pure brittle constitutive law. They are easily modeled by specifying the cross-sectional area ($A_{f}$ for the fabric and $A_{m}$ for the matrix) and the parameters for uniaxial material laws, i.e., the elastic modulus ($E_{f}$ and $E_{m}$, respectively) and ultimate tensile strain ($\varepsilon_{f,u}$ and $\varepsilon_{m,u}$, respectively), as resumed in the Figure 3. In particular, the ultimate tensile strain for mortar is defined as function of the tensile strength $f_{m,t}$, assuming 50% flexural strength $f_{m,f}$. In the absence of detailed indications, the elastic modulus assumed for mortar is calculated according to the following expression proposed in the Italian Code NTC18 [29] for the concrete in compression:

(1) $$E_{m}=22,000{\left(\frac{f_{m,c}}{10}\right)}^{\frac{1}{3}}$$

where $f_{m,c}$ is the compressive strength of the mortar. This assumption is certainly a simplification (i.e., the elastic modulus of the mortar in tension is assumed to be equal to that under compression), but it can be accepted for low values of tensile stress. Whereas the brittle hinges are modeled as zero-length rigid elements, such that we simply assign a value for the maximum stress $f_{h,t}$ so that it is equal to the cracking strength of the mortar $f_{m,t}$. They fail when the tensile strength of the mortar was reached, simulating the crack opening when the axial stress in the element drops to zero.

The fabric-matrix interface is then defined with the introduction of discrete shear springs with linear brittle behavior, as explained in the following section.

The model is loaded at one of the free fiber ends, while the other is considered to be restrained. This assumption was made coherently with the hypothesis that the first cracks appear at the extremities of the FRCM strip, close to the gripping area, as observed in several experimental studies. The non-linear static analysis is performed under a displacement controlled mode, and the axial displacement is applied to the rightmost fiber node and is increased until it reaches the target displacement. The Newton–Raphson algorithm is assumed for the solution of the non-linear system of equations, and for each step of the analysis, the output results are stored inside each node and element object, in terms of displacements and reaction forces, to simplify the post-processing analysis for both global and local responses.

### 2.2. Calibration of Interface Parameters

**Figure 4:**
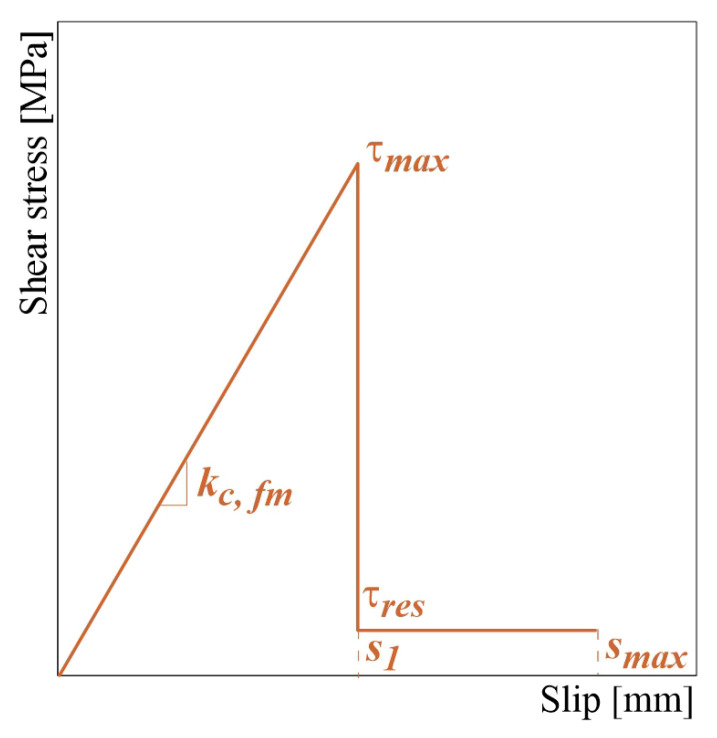
Constitutive law assumed for the fabric-matrix interface.

The fabric-matrix interface plays a fundamental role in the tensile behavior of the FRCM strip tested in tension, significantly affecting the performance and the mode of failure. However, it is very difficult to evaluate the interface properties experimentally. In this work, the fiber-mortar interface is modeled with a row of discrete shear springs with a multi-linear fragile behavior, as defined by the assumption of three parameters: the discrete value of the stiffness $K_{D,fm}$, the maximum shear stress value $\tau_{max}$ and the residual shear strength $\tau_{res}$. In this case, the discrete value of the stiffness $K_{D,fm}$ is deduced from the continuous value $k_{C,fm}$, which is the slope of the first ascending branch of the shear stress-slip law assumed at the fabric-matrix interface (see Figure 4). Assuming the shear stress is constant along two successive interface springs, the relation between $K_{D,fm}$ and $k_{C,fm}$ is defined follows:

(2) $$K_{D,fm}=k_{C,fm}b\frac{L_{FRCM}}{n}$$

where *b* is the width of the FRCM strip and *n* is the number of elements with which the model is discretized.

The value $k_{C,fm}$ is calculated assuming the empirical formulation proposed by Minafò et al., 2022 [30] for Basalt-FRCM systems:

(3) $$k_{C,fm}=0.827\omega +1.251$$

where ω is the mechanical ratio of the fabric, defined as follows:

(4) $$\omega =\frac{A_{f}f_{f}}{A_{m}f_{m,t}}$$

The maximum slip $s_{max}$ is assumed equal to the average maximum value of displacement achieved during the tensile test. Finally, the values of shear stress $\tau_{max}$ and $\tau_{res}$ are evaluated by attempting to find the best-fitting response.

It should be noted that the brittle constitutive laws for the matrix and fabric-matrix interface are simplified; the post-peak softening is very important in the evaluation of fiber-bridging effects on cracking behavior. However, the choice of brittle constitutive laws for the matrix and fibre-to-matrix interface was made due to the fact that the two constituent materials are being modeled separately and therefore their interaction is simulated through the shear spring elements. Consequently, the bridging effect offered by the fabric is automatically taken into account in the global response. It is clear that the best choice would be to introduce a linear softening constitutive law in tension for the matrix and the interface, but it implies the need to calibrate a further unknown parameter. Additionally, the choice made on the pure brittle law is consistent with the literature [15], which demonstrates that this assumption allows a good estimation of the maximum load. Further investigations will be performed that consider a more refined law.

### 2.3. Shear-Bond 1D Model

**Figure 5:**
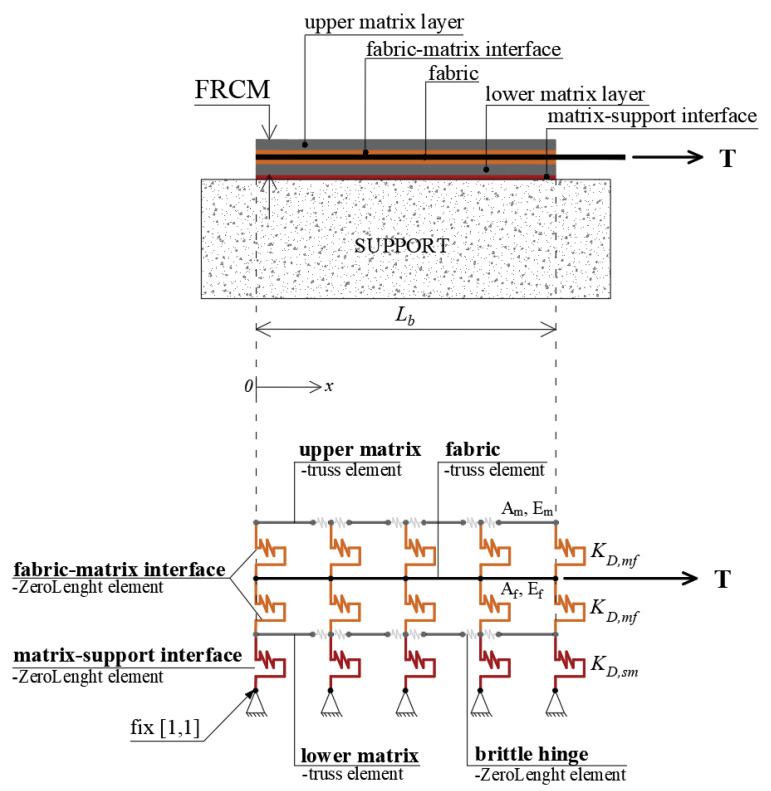
1D numerical models for shear bond behavior.

The modeling approach proposed for the tensile behavior is then extended to the case of shear-bond behavior. In this case, a further row of shear springs is introduced in order to take into account the composite-to-support interaction. Moreover, the two layers of mortar (i.e., the upper mortar layer and lower mortar layer) are modeled separately, as shown in Figure 5.

The FRCM strengthening system is modeled using three rows of truss elements, of which two are for mortar and one is for fabric, connected to each other by shear springs. Additionally, brittle hinges connect in series to the ends of each matrix truss. Under the assumption that the substrate is infinitely rigid and properly constrained, the lower mortar layer is connected to constrained nodes through shear springs simulating the reaction of the support. Furthermore, uniaxial material laws are assumed for both the fabric and mortar truss elements, simply by assigning the elastic modulus ($E_{f}$ and $E_{m}$) and the corresponding ultimate strain values ($\varepsilon_{f,u}$ and $\varepsilon_{m,u}$). Two rows of discrete shear springs simulate the upper/lower mortar-fabric interface. These are characterized by the assumed parameters (i.e., $k_{C,fm}$, $\tau_{max}$ and $\tau_{res}$) previously calibrated for tensile behavior. For the sake of simplicity, a linear-elastic law is assumed at the matrix-to-support interface. The assigned shear stress-slip law is defined by the ultimate slip value $s_{max,ms}$ and the discrete value of stiffness $K_{D,ms}$ obtained by multiplying the continuous value $k_{C,ms}$ by the bond area between two successive shear springs at the matrix-support interface.

## 3. Reference Experimental Tests

The two proposed models are validated against the experimental benchmarks from the literature, Lignola et al. [31], referred to as the tensile and shear bond tests on Basalt-FRCM applied on clay brick masonry.

The experimental results are referred to as the tensile and shear bond tests on three different basalt-based FRCM configurations, namely “FRCM1”, “FRCM2” and “FRCM4”. The tests are performed by different European laboratories: University Claude Bernard Lyon (unilyon), Cracow University of Technology (cut), University of Chieti (unich), University of Naples “Federico II” (unina) and University of Sannio (unisannio). The textile-matrix combinations are outlined in Table 1 (rewritten from [31]), specifying the corresponding mechanical properties for both fiber and mortar, which have been experimentally evaluated by single laboratories.

The specimens from sets FRCM1 and FRCM2 consist of a coated-basalt-fiber grid with a unit weight of 220 g/m^2^ and a yarn cross-sectional area of 0.83 mm^2^. embedded between two layers of cement and lime-based mortar, respectively. Whereas, specimens of the set FRCM4 are made of lime-based mortar and a coated-basalt-fiber grid, with a unit weight of 250 g/m^2^ and a yarn cross-sectional area of 0.23 mm^2^.

The tensile behavior is evaluated on specimens with various dimensions. In particular, the width of the strip ranges from 75 to 100 mm, and the length ranges from 500 to 650 mm. More details about the dimensions of the FRCM tensile specimens are reported in Table 2.

Tensile tests are carried out in a displacement control mode at slightly different rates for each set of specimens, from 0.25 mm/min for FRCM1 (unich) up to 1.0 mm/min for FRCM4 (unina) and FRCM4 (unisannio). The experimental results in terms of stress-strain curves show that the typical tri-linear trend is not always observed due to the adhesion properties established at the fiber-mortar interface, as explained in the following sections. Moreover, different modes of failure are observed, in particular: slippage in the clamping area before crack formation or tensile fiber rupture; cracking close to the clamping area; debonding at the interface followed by fiber slippage.

Then, the shear bond behavior is evaluated on the three sets of FRCM applied masonry prisms made of clay bricks. In this case, the bond length is equal to 260 mm and the bond width varies between 50 and 125 mm. The specimen dimensions are listed in Table 3.

Shear-bond tests are performed by applying the load at the un-bonded fiber strip, assuming the displacement control mode at slightly different rates for each set of specimens. The results in terms of stress-slip curves show significant differences due to the amount of variables involved in the test set-up, such as the use of different types of displacement transducers and different configurations for the measurement instrumentation. The main mode of failure observed is the tensile fiber rupture, and in some cases it is combined with debonding at the fiber-mortar interface or with fiber slippage.

## 4. Numerical Results

As observed experimentally, the constitutive behavior of FRCM composites strictly depends on the mechanical properties of the material constituents, as well as the test set-up and the manufacturer of the samples. Moreover, a strong relation is observed between the mechanical response and the fabric-matrix interaction.

In this work, the parameters assumed for the fabric-matrix and matrix-support interface are calibrated on the basis of the experimental response from tensile and shear-bond tests, respectively. The interface parameters are listed in Table 4.

The two stress values (i.e., the maximum and the residual stress) were calibrated on the basis of the experimental results, taking into account the failure mode affecting the single sample and assuming higher or lower values if the test ended as soon as decontamination or breakage of the fiber occurred.

### 4.1. Validation of the Tensile 1D Model

**Figure 6:**
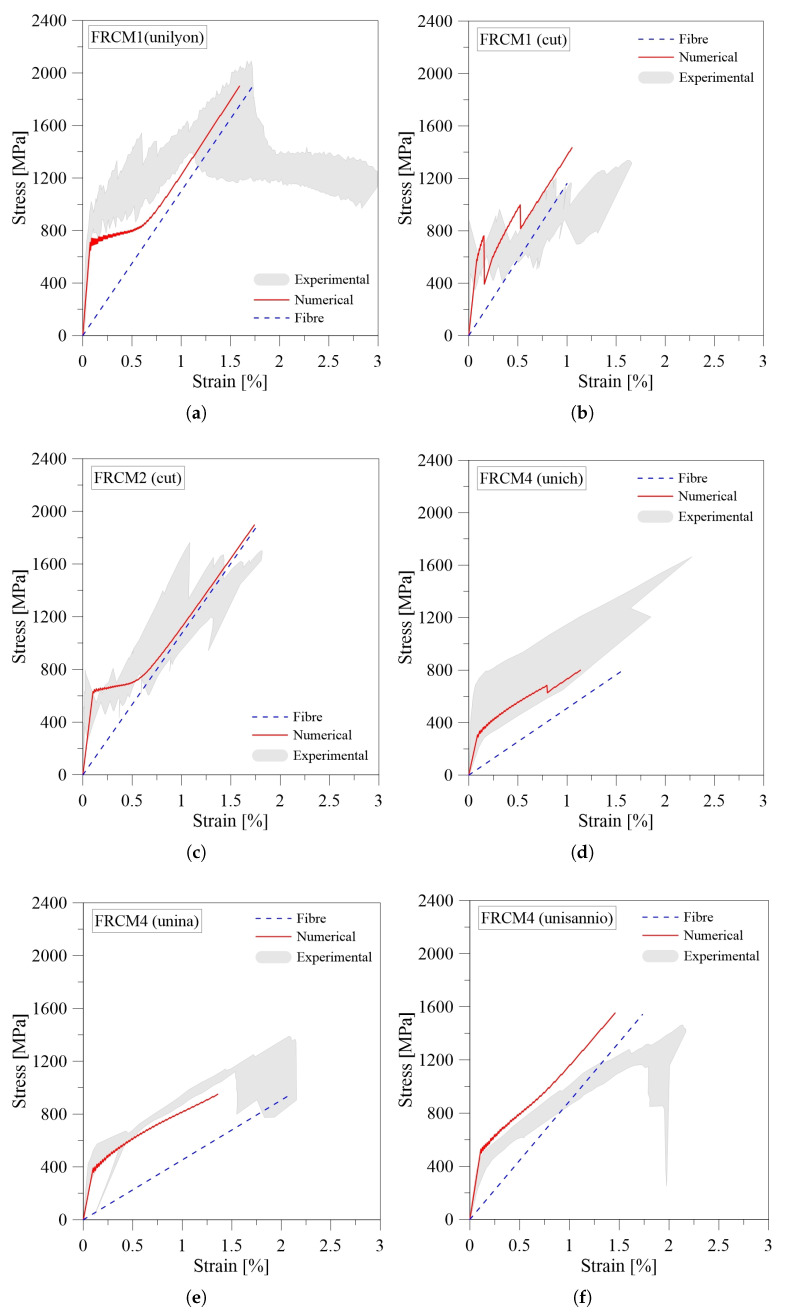
Numerical-experimental comparison of tensile behavior for Basalt-FRCM: (**a**) FRCM1 (unilyon); (**b**) FRCM1 (cut); (**c**) FRCM2 (cut); (**d**) FRCM4 (unich); (**e**) FRCM4 (unina); (**f**) FRCM4 (unisannio).

The numerical results from tensile 1D model are compared in terms of the stress-strain curves with the experimental benchmarks from the literature [31] (Figure 6). As discussed above, the parameters investigated are the dimensions of the FRCM strip and the mechanical properties of the matrix and fabric.

The comparisons were useful to calibrate the stress-slip laws assumed at the fabric-matrix interface, in particular the shear stress values $\tau_{max}$ and the residual shear stress $\tau_{res}$. Numerically it has been observed that the value of $\tau_{max}$ mainly affects the first ascending branch of the curve, whereas $\tau_{res}$ affects the second and the third phases of the curve.

Observing the curves, the proposed tensile model reproduces the experimental trend well. The first un-cracked stage is mainly governed by the mechanical properties of the mortar (elastic modulus and tensile strength), whereas the post-cracking phases strictly depend on the fabric-matrix interlocking.

It is worth pointing out that the typical tri-linear trend of FRCM composites tested in tension is not always observed. In particular, only samples FRCM1 (unilyon) (Figure 6a) and FRCM2 (cut) (Figure 6c) exhibit a behavior that may be considered similar to a tri-linear curve, characterized by different values of stiffness. This response is clearly linked to the stress–slip relationship adopted at the fabric-matrix interface and in particular to the $\tau_{res}$ value that rules the second and third branches of the curve. When the value for $\tau_{res}$ is low, fiber slippage occurs and the mortar does not contribute to the load transfer in the third stage, as is also observed experimentally [31]. In the other cases, a more bi-linear behavior is obtained numerically. The $\tau_{res}$ value is assumed to be about an order of magnitude greater than in the previous cases, thus after reaching the maximum shear stress $\tau_{max}$, the fiber and mortar continue to interact through a constant stress resulting from friction at the interface level. In some cases, i.e., FRCM4 (unich) (Figure 6d) and FRCM4 (unina) (Figure 6e), the model is unable to reach the maximum strain that is experimentally observed. This can be attributed to the uncertainty in the evaluation of the mechanical properties for the fiber grid.

It is observed that the decaying phase cannot be properly predicted with this type of constitutive law assumed for the interface. However, it should be noted that FRCMs are usually characterized in tension by a tri-linear relationship with the final hardening branch, and consequently the model can be considered valid for most of the cases.

### 4.2. Validation of the Shear-Bond 1D Model

**Figure 7:**
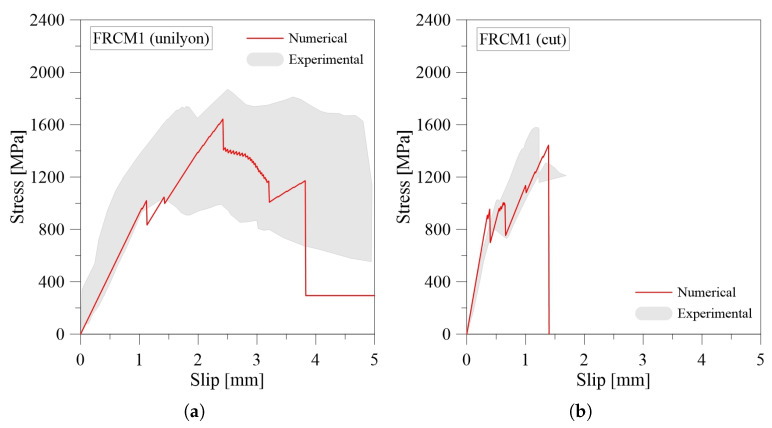

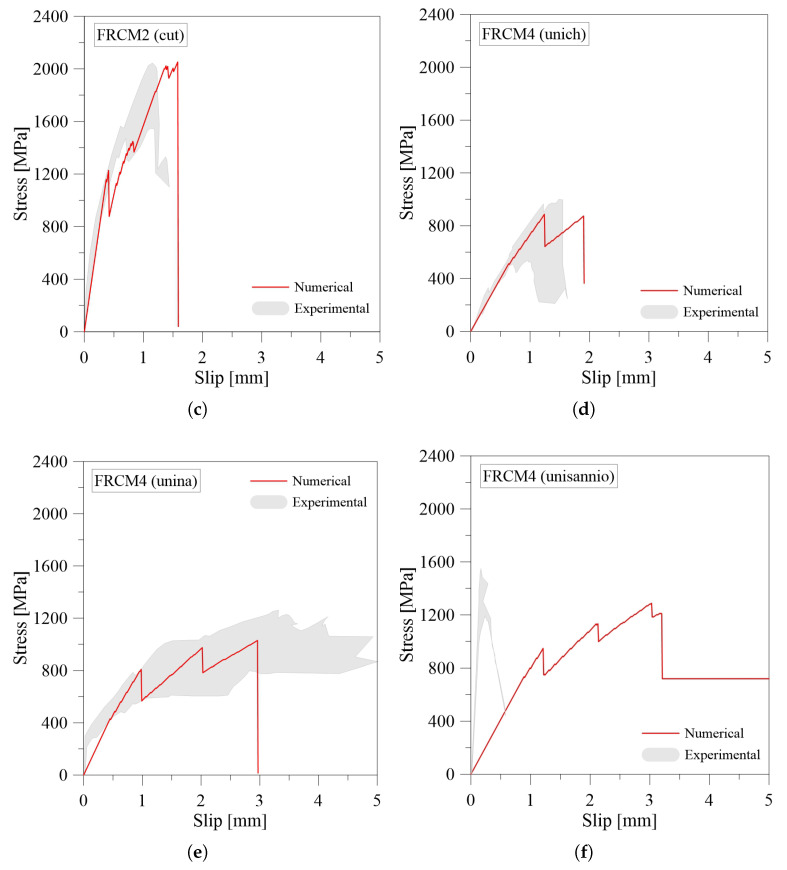
Numerical-experimental comparison of shear-bond behavior for Basalt-FRCM: (**a**) FRCM1 (unilyon); (**b**) FRCM1 (cut); (**c**) FRCM2 (cut); (**d**) FRCM4 (unich); (**e**) FRCM4 (unina); (**f**) FRCM4 (unisannio).

Figure 7 shows the comparison between the benchmark experimental data [31] and the numerical results of the shear-bond 1D Model. From the comparisons, it is possible to observe that the numerical and experimental curves are in good agreement. In particular, the model is able to reproduce the initial ascending branch up to the load-carrying capacity, where the fiber grid begins to debond from the embedded mortar layers at the loaded extremity. Moreover, the model is able to reproduce the post-peak phase with good approximation, due to the constitutive behavior assumed at the fabric-matrix interface. The shear stress values $\tau_{max}$ and $\tau_{res}$ assumed for the fabric-matrix interface have a certain influence, as well as the maximum shear stress achieved at the matrix-support interface. This was last calibrated according to the failure mode experimentally observed. In particular, higher values of $\tau_{res}$ are associated with a failure mode due to the tensile rupture of the textile. In these cases, the absence of the post peak-phase is observed and small slip values were achieved, often less than 2 mm, as shown in Figure 7b–d. However, lower values of $\tau_{res}$ are associated with those samples that fail due to fiber slippage. In these cases, during the experimental test, the obtained values of slip are greater than in the other cases, as shown in Figure 7a,e.

With regards to the numerical-experimental comparison reported in Figure 7f, a significant difference is observed between the numerical and experimental response. It is due to the fact that the slip values are experimentally evaluated with a different measurement system. It is worth pointing out that the model is able to predict the maximum strength. Indeed, the stress values from the numerical simulation are close to the values derived experimentally.

In general, the peak load numerically obtained is close to the average strength experimental value. In terms of slips, the differences are less than 15%, with the exception being sample FRCM4 (unina), as seen in Figure 7e, where the premature failure occurred due to the uncertainties related to the material properties.

## 5. Conclusions

This work focused on the mechanical behavior of FRCM composites. In particular, the influence of some selected variables were investigated: the mechanical properties of a fiber grid, the type of mortar (cement- or lime-based), the specimen dimensions and the bonded width of the FRCM systems on support. Two simple 1D models were proposed for simulating the tensile and shear behavior, respectively. The validation of the proposed models was achieved by comparing the numerical results with the experimental benchmarks from the literature. From the comparisons, it emerged that the interface constitutive laws play a key role in the global response of the specimens. The interface parameters were calibrated on the basis of the numerical tensile response by finding the values that provide the best-fitting response.

In general, the numerical model is able to reproduce the observed experimental tensile and shear bond behaviors with good approximation. Both the tensile and shear-bond 1D model are able to reproduce the experimental trend well, especially in terms of peak stress, showing a certain sensitivity to the parameters investigated, in particular to the fiber-mortar combination. Indeed, in the case of tensile behavior, the expected tri-linear behavior is not always observed due to some circumstances mainly related to the fabric-matrix interaction, which is affected by the compatibility established between the two materials that are in contact with each other. The proposed models may be considered reliable and simplified devices based on few variables, which are easily deduced. However, other comparisons with experimental results would be necessary in order to achieve a proper calibration of the interface parameters.

## Figures and Tables

**Table 1:** Mechanical properties of material constituents.

FRCM System	Fabric				Matrix		
**ID**	**Yarn Cross-Section** **[mm^2]^**	**Unit Weight** **[g/m^2^]**	** $E_{f}$ ** **[GPa]**	** $f_{f}$ ** **[MPa]**	**Type**	** $f_{m,f}$ ** **[MPa]**	** $f_{m,c}$ ** **[MPa]**
FRCM1 (unilyon)	0.83	220	111.5 *	1669 *	cement-based	4.3	16.3
FRCM1 (cut)	0.83	220	111.5 *	1669 *	cement-based	4.6	14.8
FRCM2 (cut)	0.83	220	111.5 *	1669 *	lime-based	5.8	16.2
FRCM4 (unich)	0.23	250	51	802	lime-based	6.2	15 **
FRCM4 (unina)	0.23	250	45.3	940	lime-based	6.2	15 **
FRCM4 (unisannio)	0.23	250	89 **	1542 **	lime-based	6.2	12.4

* average value. ** data sheet value.

**Table 2:** Dimensions of the FRCM tensile specimens.

FRCM System	Length	Width	Thickness	Fiber Cross-Section
ID	[mm]	[mm]	[mm]	[mm^2^]
FRCM1 (unilyon)	650	90	10	2.49
FRCM1 (cut)	584/587	75/77	10/11.6	2.49
FRCM2 (cut)	581/588	75/77	10/11.8	2.49
FRCM4 (unich)	500	100	10	3.89
FRCM4 (unina)	600	100	10	3.89
FRCM4 (unisannio)	500	100	10	3.89

**Table 3:** Dimensions of the FRCM shear-bond test specimens.

FRCM System	Bond Length	Bond Width	FRCM Thickness	Fibre Cross-Section
ID	[mm]	[mm]	[mm]	[mm^2^]
FRCM1 (unilyon)	260	90	10	2.49
FRCM1 (cut)	260	75	10/11.6	2.49
FRCM2 (cut)	260	75	10/11.8	2.49
FRCM4 (unich)	260	100	10	3.89
FRCM4 (unina)	260	125	10	3.89
FRCM4 (unisannio)	260	125	10	3.89

**Table 4:** Interface parameters.

	FABRIC-MATRIX				MATRIX-SUPPORT	
**FRCM System**	$k_{C,\mathrm{fm}}$	$\tau_{\max}$	$\tau_{\mathrm{res}}$	$s_{\max}$	$k_{C,\mathrm{ms}}$	$s_{\max,\mathrm{ms}}$
**ID**	**[N/mm^3^]**	**[MPa]**	**[MPa]**	**[mm]**	**[N/mm^3^]**	**[mm]**
FRCM1 (unilyon)	3.27	0.60	0.01	7.55	0.12	2.08
FRCM1 (cut)	2.82	0.60	0.20	2.56	0.80	0.75
FRCM2 (cut)	3.02	0.80	0.01	2.72	1.50	0.73
FRCM4 (unich)	2.37	0.40	0.10	3.71	0.15	2.33
FRCM4 (unina)	2.14	0.45	0.10	4.44	0.20	1.25
FRCM4 (unisannio)	2.85	0.70	0.10	3.55	0.15	1.30

## Data Availability

Data regarding the current study are available under request.
